# The Use of Topiramate for Weight Loss Causing Acute Glaucoma: A Case Report and Literature Review

**Published:** 2019

**Authors:** Bruno Mauricio Rodrigues DE OLIVEIRA, Pedro Vanalle FERRARI, Bruno Torres HERRERIAS, Flávio Eduardo HIRAI, Carolina Pelegrini Barbosa GRACITELLI

**Affiliations:** 1 Department of Ophthalmology, Federal University of São Paulo, São Paulo, Brazil; 2 Glaucoma Unit, Ver Mais Oftalmologia, São Paulo, Brazil

**Keywords:** Topiramate, Angle-Closure Glaucoma, Ciliochoroidal effusion, Myopic shift, Weight loss

## Abstract

Topiramate is a sulfa-containing drug which is able to disrupt the ocular blood barrier. Recently it has gained more popularity, being used in many clinical conditions. Nowadays, the cases of glaucoma induced by topiramate have increased due to the use of this drug to induce weight loss. We here described a 29-year-old female presented with a one-day history of blurred vision in both eyes and headache. She was using a weight loss formula containing topiramate 100 milligrams. Ophthalmologic exam revealed an important myopic shift of -7.00 spherical diopters at presentation with intraocular pressure (IOP) of 32 mmHg and a shallow anterior chamber in both eyes. After discontinuous of topiramate and use of cycloplegic eyedrops, myopic shift improved and IOP controlled after two days. The anterior chamber was significantly deeper in both eyes after two weeks. It is theorized that topiramate can provoke a ciliochoroidal effusion and, therefore, can cause an anterior displacement of lens-iris diaphragm with a secondary angular closure. The treatment must include cycloplegic and discontinuation of the drug. Sulfa-containing drugs lead to an indirect mechanism of angle closure, frequently bilateral and, as mentioned above, with a different treatment approach. If unrecognized and untreated, it can provoke high morbidity with possibility of bilateral permanent visual loss.

## INTRODUCTION

Glaucoma is the most common form of optic neuropathy described as progressive loss of retinal ganglion cells (RGCs) as well as typical changes to the retinal nerve fiber layer (RNFL) and optic nerve [1, 2]. Depending on the angle presentation at gonioscopy, it is classified as open-angle or closed-angle glaucoma. Also, if an underlying cause is identified, it is recognized as secondary glaucoma. Considering closed-angle glaucoma features, secondary drug-induced is a rare entity with a high potential of permanent visual loss. Medications have two mechanisms to produce acute closed-angle glaucoma, a direct effect due to pupillary dilation or an indirect effect due to blood-ocular barrier disruption and choroidal and ciliary body effusion. For the second mechanism, sulfa-containing drugs are the most related medication class, including antibiotics (trimethoprim-sulfamethoxazole), diuretics (hydrochlorothiazide, acetazolamide and furosemide), rheumatologic drugs (sulfasalazine) and other drugs like topiramate, chlorpropamide, and sumatriptan [3, 4]. Topiramate is a sulfamate-substituted monosaccharide with large use in general medicine. It was first developed to be an antiepileptic medication, but recently it has gained more popularity, being used to treat migraine, depression, neuropathic pain and also for weight loss. In 2001, it was recognized that acute closed-angle glaucoma is a certain adverse side effect of the drug [5]. Nowadays, the cases of glaucoma induced by topiramate have increased due to the use of this drug to prevent migraine or induce weight loss [6-9]. 

In this article, we illustrate one case of topiramate-induced bilateral acute angle closure glaucoma and myopic shift. Also, we discuss some management controversies and provide literature review about this entity. 

## CASE REPORT

**Figure 1:**
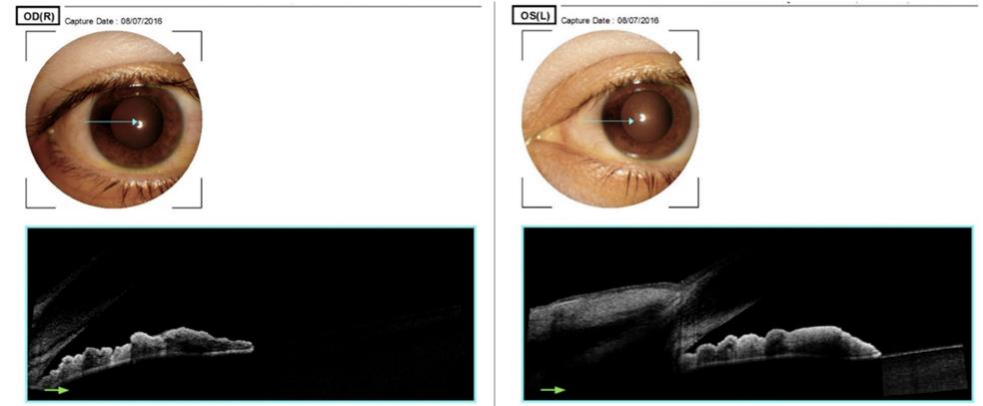
Anterior segment opticalCoherence Tomography (OCT) Showing Bilateral Appositional Angle Closure. OD: Right Eye; OS: Left Eye.

**Figure 2:**
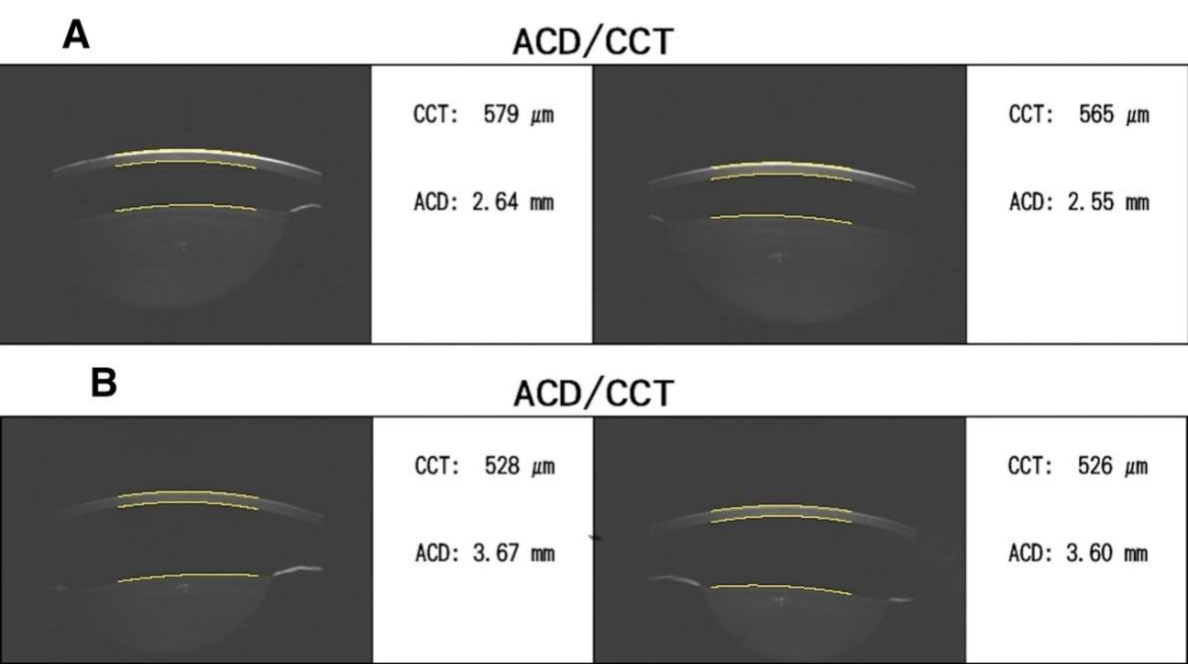
Anterior Chamber Depth (ACD) Evaluation at Admission (2A) and after Two Weeks of Treatment (2B). CCT: Central Corneal Thickness; µm: micrometer.

This is a case report of a 29-year old Caucasian woman evaluated in 2016. The present study was conducted in glaucoma clinics at Federal University of São Paulo. A written informed consent was obtained from the patient. Also, it was performed in accordance with the tenets of the Declaration of Helsinki. The patient presented with a one-day history of decreased visual acuity in both eyes (OU) described as blurring of vision and associated with headache. She denied any personal, ophthalmological, and family medical history. She was using a weight loss formula containing one-hundred milligrams of topiramate that she started a few days before. Ophthalmologic examination on admission revealed a count fingers visual acuity that improved to 0.4 logMAR in OU with refraction of -7.00 spherical diopters in OU. Her previous spectacles prescription was of -2.25 cylindrical diopters at 180 degrees in the right eye (OD) and -2.75 cylindrical diopters at 180 degrees in the left eye (OS). Slit lamp exam demonstrated a bilateral shallow anterior chamber (AC) without synechiae or inflammation signs. The pupillary reflex was normal in OU. The intraocular pressure (IOP) was 32 mmHg in OU. Gonioscopy revealed closed angle in OU. The pachymetry exam was 579 micrometer (µm) in OD and 565 µm in OS. On fundoscopy, she had a cup to disc ratio of 0.5 in OD and 0.4 in OS. Anterior segment optical coherence tomography (OCT) revealed an appositional angle closure (Fig 1) and AC measurements of 2.64 millimeters (mm) in the OD and 2.55 mm in the OS. The pachymetry exam was 579 micrometer (µm) in OD and 565 µm in OS. (Fig 2A).

The treatment was started with tropicamide 1% every 8 hours in OU as well as timolol maleate 0.5% and brinzolamide 1% every 12 hours in OU. Topiramate formula was also discontinued. After 40 minutes of cycloplegic instillation, the myopic shift decreased to -4.00 spherical diopters and the IOP decreased to 22 mmHg in OU. The patient returned for revaluation after two days with visual acuity improvement. Her myopic shift decreased, presenting with refraction of -1.00 spherical diopter and visual acuity of 0.1 logMAR in OU. The IOP decreased to 5 mmHg in OD and 9 mmHg in OS. After that, we discontinued the timolol maleate and brinzolamide eyedrops. 

After two weeks, her corrected distance visual acuity (CDVA) was 0 logMAR in OU with refraction of -1.00 spherical diopter and -2.75 cylindrical diopters at 175 degrees in OD and -0.50 spherical diopter and -2.50 cylindrical diopters at 175 degrees in OS. The IOP was 13 mmHg in OD and 14 mmHg in OS. The AC measurements improved to 3.67 mm in OD and 3.60 mm in OS. The pachymetry exam was 528 µm in OD and 526 µm in OS. (Fig 2B).

## DISCUSSION

We here described a 29-year-old female presented with a one-day history of blurred vision OU and headache. She was using a weight loss formula containing topiramate 100 milligrams. She was developed myopic shift of -7.00 spherical diopters at presentation with IOP of 32 mmHg and bilateral shallow AC. After discontinuous of topiramate and use of cycloplegic eyedrops, myopic shift improved and IOP controlled after two days. The AC was significantly deeper in both eyes after two weeks.

In 2001, Banta et al. [5] were the first to describe a bilateral angle closure glaucoma and myopic changes caused by topiramate use. With the advancement of ocular ultrasound technology, it was possible to evaluate the underlying mechanism of ciliochoroidal effusion provoked by sulfa-containing drugs [5, 10, 11]. It is theorized that disruption of ocular blood barrier caused by these drugs induces a ciliochoroidal effusion and anterior rotation of the ciliary body [5, 10, 11]. Therefore, anterior displacement of lens-iris diaphragm provokes angle closure and relaxation of the zonules cause lens thickening with a myopic shift induction [5, 10, 11].

Hence, according to this mechanism, myotic drugs and iridectomies are not effective and should not be performed [12, 13]. Fraunfelder et al. [12] reviewed 115 reports of topiramate-induced angle closure glaucoma and found that 38% of patients had a laser or surgical iridectomies indicating confusion with pupillary block, narrow-angle glaucoma entity. It is estimated that topiramate induces a five-fold risk of closed-angle glaucoma in patients younger than 50 years old [14].

The correct management is immediately discontinuous of topiramate and prescription of cycloplegic and antihypertensive eyedrops [3, 5, 6, 8, 12, 13]. During the follow-up, we can evaluate refraction, managing the myopic shift as well as the IOP and AC depth. Myotic agents such as pilocarpine should be avoided because cholinergic agents may worsen the lens-iris diaphragm displacement [3, 12, 13]. In addition, some reports suggest that the use of high doses of intravenous corticosteroids at the time of presentation can be beneficial [6, 15, 16]. Ultrasonography images can evaluate the ciliochoroidal effusions, helping to diagnose this drug-related entity [10]. Anterior segment OCT is a very useful tool that permits evaluation of AC depth, iris-lens configuration and angle disposition [17, 18]. Optical coherence tomography is a noninvasive method that provides high-quality images of the anterior segment. The images can be analyzed and measurements of the AC and angle can be obtained and used for follow-up purposes. Previous reports recognize the importance of OCT to establish the diagnosis and guide the follow-up and therapeutic response, avoiding unnecessary interventions [17, 18]. In our case, it was possible to follow the AC depth increase of more than one mm in both eyes after the treatment was instituted. Ultrasound biomicroscopy can evaluate the same features as OCT, but requires cooperation by the patient, causes some discomfort and is operator-dependent [19].

As this clinical condition is better understood, many other ocular features are recognized and related to topiramate. Recent studies suggest serous retinal detachment, macular striae, anterior uveitis and panuveitis as possible associated conditions [20-24]. There is one report of acute myopia and glaucoma in a 7-year-old child [25]. Also, it is well established in the literature that the ciliochoroidal effusion syndrome provoked by topiramate is an idiosyncratic reaction because it is a rare event [8, 12, 26], it is not dose-dependent [8, 27] and there are controversies reports about rechallenge drug tests [8, 12, 26]. One recent study also demonstrated that the time of onset of symptoms can be variable, reporting a patient who developed acute glaucoma after 262 days of topiramate use [28].

## CONCLUSIONS

In summary, many drugs can lead to secondary glaucoma. Most drug-related events of acute angle-closed glaucoma are related to pupillary block induced by sympathetic drugs or parasympathetic inhibitors. However, sulfa-containing drugs lead to an indirect mechanism of angular closure, frequently bilateral and, as mentioned above, with a different treatment approach. If unrecognized and untreated, it can provoke high morbidity with possibility of bilateral permanent visual loss.
